# Immediate versus staged revascularization in acute coronary syndrome and multivessel disease: a meta-analysis and meta-regression of RCTs

**DOI:** 10.1007/s11739-026-04369-z

**Published:** 2026-05-12

**Authors:** Felix Bergmann, Anselm Jorda, Theresa Pecho, Lukas Stoiber, Georg Gelbenegger, Lena Pracher, Amelie Leutzendorff, Gregor Heitzinger, Jolanta M. Siller-Matula, Christian Schörgenhofer, Markus Zeitlinger, Irene Lang, Valentin al Jalali

**Affiliations:** 1 Department of Clinical Pharmacology, Medical University of Vienna, Waehringer Guertel 18-20, 1090 Vienna, Austria; 2 Department of Medicine II, Division of Cardiology, Medical University of Vienna, Vienna, Austria; 3 Department of Experimental and Clinical Pharmacology, Centre for Preclinical Research and Technology (CEPT), Medical University of Warsaw, Warsaw, Poland

**Keywords:** Myocardial infarction, ACS, Intervention, Angiography, Angioplasty, PCI

## Abstract

In patients presenting with acute coronary syndrome (ACS) and multivessel disease, it is unclear whether non-culprit lesions should be revascularized immediately or in a staged procedure. We performed a meta-analysis of randomized controlled trials that compared immediate versus staged revascularization strategies. Two authors independently screened records from the online databases PubMed, Embase, Web of Science, and the Cochrane Library to identify eligible trials up to November 2025. The primary outcome was all-cause mortality at 1 year. Secondary outcomes included cardiovascular events at 1 year. Meta-regressions were performed to explore the influence of study-level characteristics. This study was registered in PROSPERO (CRD42023446181). Ten trials with a total of 5651 patients were included in the final analysis. All-cause death at 1 year occurred in 109 (3.9%) of 2809 patients in the immediate revascularization group and 100 (3.5%) of 2842 patients in the staged revascularization group (risk ratio, 1.10; 95% CI 0.79–1.52). Cardiovascular death at 1 year occurred in 76 (2.8%) of 2749 patients in the immediate revascularization group and 66 (2.4%) of 2768 patients in the staged revascularization group. Meta-regression suggested that higher mean patient age was significantly associated with a lower risk ratio favoring the immediate revascularization group (regression coefficient -0.10; 95% CI -0.16 to -0.002; p = 0.047). All-cause mortality and cardiovascular deaths were similar in patients with ACS, predominantly with STEMI, and multivessel coronary disease undergoing immediate or staged revascularization. The available effect estimates do not exclude the possibility of clinically meaningful benefit or harm from immediate revascularization.

**Supplementary Information:**

The online version contains supplementary material available at 10.1007/s11739-026-04369-z.

## Introduction

Acute coronary syndrome (ACS) is a medical emergency that usually requires immediate or early percutaneous coronary intervention (PCI) to revascularize the culprit coronary artery [1]. Multivessel disease is a frequent condition in patients presenting with ACS [2]. Previous randomized controlled trials have confirmed that complete revascularization of non-culprit lesions is superior to a culprit lesion-only strategy in these vulnerable patients [3–6]. The COMPLETE trial demonstrated that patients with ST-elevation myocardial infarction (STEMI) and multivessel coronary artery disease had a lower risk of major adverse cardiovascular events after complete revascularization compared to PCI of culprit-lesions only [3]. However, it is unclear whether revascularization of non-culprit lesions should be performed immediately during the index procedure or in a staged procedure. This uncertainty is also highlighted by contemporary guidelines from the American College of Cardiology and the European Society of Cardiology [7–10]. To investigate this gap in evidence, a meta-analysis comprising more than 40,000 patients, mainly from retrospective studies, found that immediate complete revascularization of non-culprit lesions was associated with higher short- and long-term mortality compared with staged PCI [11]. The recently published randomized controlled trial OPTION-STEMI failed to demonstrate non-inferiority of the immediate revascularization strategy [12]. In contrast, two randomized controlled trials (BIOVASC and MULTISTARS AMI) showed non-inferiority of immediate revascularization, with a trend toward superiority of the immediate revascularization strategy [13, 14]. However, in another recent randomized controlled trial (iMODERN), immediate revascularization was not superior to staged revascularization [15].

Due to these conflicting findings, we conducted a systematic review and meta-analysis to summarize the available evidence from randomized controlled trials comparing an immediate with a staged revascularization strategy. This meta-analysis aimed to compare clinical outcomes of immediate versus staged revascularization strategies of non-culprit lesions in patients presenting with ACS and multivessel coronary artery disease.

## Methods

### Study inclusion and procedures

We performed a systematic review and meta-analysis of randomized controlled trials comparing immediate with staged revascularization strategies of non-culprit lesions in patients with ACS and multivessel coronary disease. Two authors independently screened records from the online databases PubMed, Embase, Web of Science, and the Cochrane Library to identify eligible trials. We considered trials published in English up to November 2025. The exact search input for each database with the corresponding number of identified records is provided in Supplementary Table 1. References of included studies and previous systematic reviews on the topic were also screened to identify additional studies. Inclusion criteria were (i) randomized controlled trials, (ii) comparing immediate with staged revascularization strategies in patients with ACS, and (iii) reporting mortality. Non-randomized trials, observational data, and other article types such as reviews or study protocols were excluded. Studies published in non-peer-reviewed journals or journals not indexed in Web of Science or PubMed were also excluded. Two reviewers (A.J. and V.J.) screened records using title, abstract, and full text to select eligible studies. In case of disagreement, a third reviewer (T.P.) was consulted to reach a consensus.

This meta-analysis was conducted according to the recommendations of the PRISMA (Preferred Reporting Items for Systematic Reviews and Meta-Analyses) statement. The PRISMA checklist can be found in the supplement. The study was registered in PROSPERO with the identifier CRD42023446181. Since this study was based exclusively on previously published data, no new procedures involving human participants or animals were conducted by the authors. Therefore, ethical approval and informed consent were not required for this work. 

### Data extraction and outcomes

The meta-analysis was based on the outcomes of the intention-to-treat population of the individual trials. Data were extracted from full-text articles and their supplemental files. No unpublished or patient-level data were accessed. Two authors extracted the data (A.J. and T.P.) using standardized forms for outcomes and baseline data of interest. Discrepancies in data extraction were clarified by personal discussion.

The primary outcome of this meta-analysis was all-cause mortality at 1 year. Secondary outcomes included cardiovascular death, subsequent myocardial infarction, cerebrovascular events, target-vessel revascularization, and major adverse cardiovascular events (MACE) or major adverse cardiovascular and cerebrovascular events (MACCE). The definitions of MACE and MACCE varied among the individual trials and are provided in Supplementary Table 2. The exact definitions of myocardial infarction as endpoint were provided by two trials (Supplementary Table 3) [13, 14].

We performed subgroup analyses based on the type of myocardial infarction (ST-elevation [STEMI] versus non-ST-elevation myocardial infarction [NSTEMI]), the time between the index and staged procedure (< 2 weeks versus ≥ 2 weeks), age, diabetes, number of nonculprit lesions, and localization of culprit lesion (left anterior descending [LAD] versus left circumflex artery [LCX]/right coronary[RCA]). Different studies employed varying age thresholds for their subgroup analyses: OPTION-STEMI and iMODERN used < 65 versus ≥ 65 years, BIOVASC used < 70 versus ≥ 70 years, while MULTISTARS used < 75 versus ≥ 75 years. Exploratory univariate meta-regression analyses were performed to assess the association between trial characteristics and effect sizes across the trials.

The certainty of evidence for the primary outcome was assessed following the Grading of Recommendations, Assessment, Development, and Evaluations (GRADE) recommendations [16]. Risk of bias in the included trials was assessed using the Cochrane Risk of Bias (RoB) tool.

### Statistical analysis

Categorical variables are reported as numbers with percentages. We calculated pooled risk ratios (RR) with 95% confidence intervals (95% CI) using the Mantel–Haenszel method. We used a random-effects model because of the methodological and statistical heterogeneity between the trials.

Statistical heterogeneity between the trials was assessed using Chi-square statistics and Higgins's I^2^ value. I^2^ values of 0–29%, 30–49%, 50–74%, and 75–100% were interpreted as minimal, moderate, high, and very high heterogeneity, respectively [17]. Subgroup differences were tested by performing a test for heterogeneity across subgroups. Sensitivity analyses included the sequential removal of each individual study and the effect of switching from a random-effects model to a fixed-effects. We reported unadjusted p values, which were considered statistically significant at a two-sided alpha of 0.05.

Exploratory univariate meta-regression analyses were performed using random-effects restricted maximum likelihood estimation. The log-transformed risk ratio of all-cause mortality at 1 year was fitted as the dependent variable of the linear model. The total sample size of the intention-to-treat population, number of trial sites, mean age of participants, proportion of male participants (%), chronic hypertension (%), diabetes (%), all-cause mortality (%), and days from primary to index procedure in the staged group were tested as independent variables. Linear regression coefficients with corresponding 95% CIs and p values are reported. P values were not adjusted for multiple testing due to the exploratory nature of the meta-regression. Multivariate meta-regression was not performed due to the limited number of studies and the risk of overfitting. All meta-regression analyses were performed with the *R* package *'metafor'*.

The statistical analysis was performed using *R* (R version 4.1.2, 2021, Vienna, Austria) and *Review Manager* (version 5.4.1, 2014, Copenhagen: The Nordic Cochrane Centre, The Cochrane Collaboration).

## Results

### Description of trials

The screening and selection process with the corresponding number of identified records is shown in the study flow chart (Supplementary Fig. 1). The final analysis included ten randomized controlled trials, which included a total of 5651 patients [12–15, 18–23]. The key trial characteristics are shown in Table 1.

**Table 1:** Key characteristics of included trials

Trial	iMODERN	OPTION-STEMI	MULTISTARS AMI	BIOVASC	Park et al. [18]	Nichita-Brendea et al. [23]	Tarasov et al. [22]	SMILE	Maamoun et al. [21]	Politi et al. [20]
Year of publication	2025	2025	2023	2023	2023	2021	2017	2016	2010	2009
Trial design	RCT	RCT	RCT	RCT	RCT	RCT	RCT	RCT	RCT	RCT
Statistical design	Superiority	Non-inferiority	Non-inferiority	Non-inferiority	Non-inferiority	Superiority	Superiority	Superiority	Superiority	Superiority
Primary outcome	MACE at 3y	MACE at 1y	MACCE at 1y	MACCE at 1y	MACE at 1y	All-cause death at 1y	All-cause death at 1y	MACCE at 1y	MACE at 1y	MACE at 1y
Regions	Netherlands, Belgium, Spain, Portugal, Luxembourg, Slovenia, Italy, Switzerland, Czech Republic, United Kingdom, Denmark, Germany, Australia, New Zealand, Thailand	South Korea	Switzerland, Germany, Albania, Austria	Belgium, Italy, Netherlands, Spain	Korea	Romania	Russia	Italy	Yemen	Italy
N of study centers	41	14	37	29	22	1	1	2	1	1
Enrollment period	12/2017–02/2022	12/2019–01/2024	10/2016–06/2022	06/2018–10/2021	10/2011–10/2016	01/2017- 06/2019	12/2013–12/2024	09/2011–08/2013	1/2007- 12/2008	01/2003–12/2007
N of ITT population	1146	994	840	1525	209	100	136	527	78	130
N of immediate group	558	498	418	764	103	50	67	264	42	65
N of staged group	588	496	422	761	106	50	69	263	36	65
STEMI (%)	100%	100%	100%	40%	100%	100%	100%	0%	100%	100%
NSTEMI (%)	0%	0%	0%	52%	0%	0%	0%	100%	0%	0%
Unstable angina (%)	0%	0%	0%	8%	0%	0%	0%	0%	0%	0%
Primary to staged procedure (days)	Median 40 (IQR 28–58)	Median 3 (IQR 2–4)	Median 37 (IQR 30–43)	Median 15 (IQR 4–28)	Mean 4 (SD 7)	2–3 (per protocol)	Mean 10 (SD 5)	Mean 5 (SD 1)	7 (per protocol)	Mean 57 (SD 13)

Data are expressed as median (interquartile range), mean ± standard deviation, or number (%)

*ITT* intention-to-treat, *IQR* interquartile range, *MACCE* major adverse cardiovascular and cerebrovascular event, *MACE* major adverse cardiovascular event, *NSTEMI* non-ST-elevation myocardial infarction, *RCT* randomized controlled trial, *SD* standard deviation, *STEMI* ST-elevation myocardial infarction

Two trials (SMILE and Politi et al.) were conducted in Italy (Europe) [19, 20]. The trial by Park et al. was conducted in Korea (Asia) [18]. The MULTISTARS AMI trial had 37 study centers across Switzerland, Germany, Albania, and Austria (Europe) [14]. The BIOVASC trial, the largest trial, was conducted in 29 hospitals across Belgium, Italy, the Netherlands, and Spain (Europe) [13]. The OPTION-STEMI trial was conducted in South Korea [12]. The iMODERN trial had 41 study centers mainly from Europe, with additional centers in Australia, New Zealand and Thailand [15]. The trials by Nichita-Brendea et al., Tarasov et al., and Maamoun et al. were single-center trials in Romania, Russia, and Yemen, respectively [21–23].

The SMILE trial exploratorily compared the outcomes between immediate and staged revascularization strategies. The trial by Politi et al. compared three study arms: (i) immediate complete revascularization, (ii) staged complete revascularization, and (iii) culprit-lesion-only revascularization. This meta-analysis included only the results of the immediate and staged revascularization strategy study arm. In contrast, the OPTION-STEMI, BIOVASC and MULTISTARS AMI trials and the trial by Park et al. were designed to show non-inferiority of immediate to staged revascularization strategies. The iMODERN trial was designed to show superiority of the immediate revascularization strategy.

The SMILE trial included only patients presenting with non-ST-elevation myocardial infarction (NSTEMI). OPTION-STEMI, iMODERN, MULTISTARS AMI and the trials by Park et al., Politi et al., Nichita-Brendea et al., Tarasov et al., and Maamoun et al. included only patients presenting with STEMI. The BIOVASC trial included all patients presenting with any type of acute coronary syndrome (i.e., ST-elevation myocardial infarction [STEMI], NSTEMI, or unstable angina). A complete list of inclusion and exclusion criteria is provided in Supplementary Table 4.

In the OPTION-STEMI and iMODERN trials, staged revascularization of non-culprit lesions was performed after a median of 3 (IQR 2–4) days and 40 (IQR 28–58) days, respectively (Table 1). In the BIOVASC and MULTISTARS AMI trials, the corresponding median times were 15 (IQR 4–28) days and 37 (IQR 30–43) days. Across earlier studies, staged procedures occurred after a mean of 57 ± 13 days in the Politi et al. trial, 5± 1 days in the SMILE trial, and 4 ± 7 days in the Park et al. trial. Supplementary Table 5 provides the baseline characteristics of study participants. Patients were on average around 65 years old. About 80% of the study participants were male. In the studies that reported the location of the culprit lesion, approximately 40% of culprit lesions each were found in the left anterior descending artery (947 [41%] of 2333 in the immediate revascularization strategy group versus 945 [40%] of 2374 in the staged revascularization strategy group) and the right coronary artery (932 [40%] of 2333 in the immediate revascularization strategy group versus 955 [40%] of 2374 in the staged revascularization strategy group) (Supplementary Table 6). According to the American College of Cardiology/American Heart Association criteria, most lesions were classified as Type C (863 [37.1%] of 2324 in the immediate revascularization strategy group versus 895 [39.1%] of 2291 in the staged revascularization strategy group) (Supplementary Table 7).

The medical treatment at discharge is provided in Supplementary Table 8. Almost all patients received aspirin (3342 [99.5%] of 3359). The most commonly used P2Y_12_ inhibitor was ticagrelor (841 [56%] of 1510 in the immediate revascularization strategy group versus 824 [54%] of 1512 in the staged revascularization strategy group), due to its extensive use in the BIOVASC and SMILE trials.

Supplementary Table 9 provides the procedural characteristics. The recently published trials used almost exclusively drug-eluting stents [12–15, 18, 19], whereas drug-eluting stents accounted for less than 10% in the trial by Politi et al., which was conducted between 2003 and 2007 [20].

### Primary outcome

**Fig. 1:**
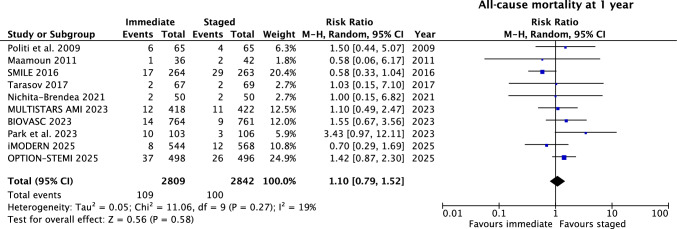
Pooled comparison of all-cause mortality at 1 year between immediate and staged revascularization strategies. *CI* confidence interval, *df* degrees of freedom, *M–H* Mantel–Haenszel method, *RR* risk ratio

In the overall analysis, the primary outcome of all-cause mortality was similar between both groups. All-cause death at 1 year occurred in 109 (3.9%) of 2809 patients in the immediate revascularization strategy group and 100 (3.5%) of 2842 patients in the staged revascularization strategy group (risk ratio, 1.10; 95% CI 0.79–1.52, p = 0.58) (Fig. 1). The I^2^ value of 19% indicated a minimal statistical heterogeneity between the studies.

### Secondary outcomes

**Fig. 2:**
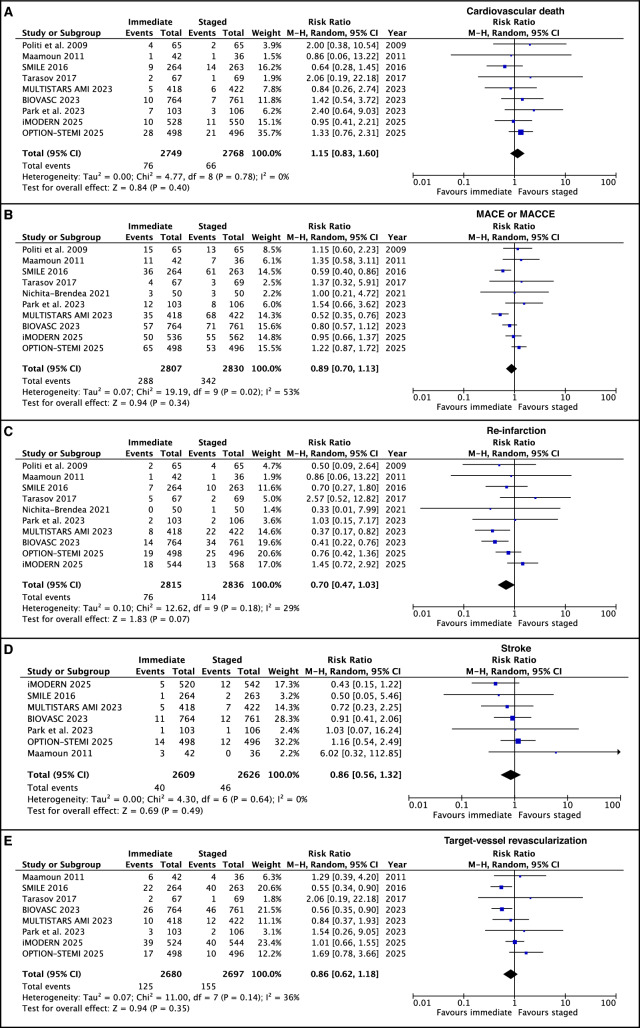
Summary estimates of secondary outcomes **A** Cardiovascular death at 1 year. **B** Any MACE or MACCE at 1 year. **C** Re-infarction at 1 year. **D** Stroke at 1 year. **E** Target vessel revascularization at 1 year. *CI* confidence interval, *df* degrees of freedom, *MACCE* major adverse cardiovascular and cerebrovascular event, *MACE* major adverse cardiovascular event, *M–H* Mantel–Haenszel method

Cardiovascular death at 1 year occurred in 76 (2.8%) of 2749 patients in the immediate revascularization strategy group and 66 (2.4%) of 2768 patients in the staged revascularization strategy group (RR 1.15; 95% CI 0.83–1.60) (Fig. 2A).

Any MACE (OPTION-STEMI, iMODERN, Park et al., Politi et al., and Tarasov et al.) or MACCE (SMILE, BIOVASC, and MULTISTARS AMI, Nichita-Brendea et al., and Maamoun et al.) occurred in 288 (10.3%) of 2807 patients in the immediate revascularization strategy group and 342 (12.1%) of 2830 patients in the staged revascularization strategy group (RR 0.89; 95% CI 0.70–1.13) (Fig. 2B).

The rate of re-infarctions did not differ significantly between the immediate revascularization strategy group (76 [2.7%] of 2815) and the staged revascularization strategy group (114 [4.0%] of 2836) (RR 0.69 [95% CI 0.46–1.03]) (Fig. 2C).

The frequency of stroke was only reported by seven of the ten trials and was similar between both groups (40 [1.5%] of 2609 versus 46 [1.8%] of 2626) (RR 0.86; 95% CI 0.56–1.32) (Fig. 2D).

Target-vessel revascularization occurred in 125 (4.7%) of 2680 of patients in the immediate revascularization strategy group and in 155 (5.7%) of 2697 patients in the staged revascularization strategy group (RR 0.86; 95% CI 0.62–1.18) (Fig. 2E).

### Subgroup analyses

**Fig. 3:**
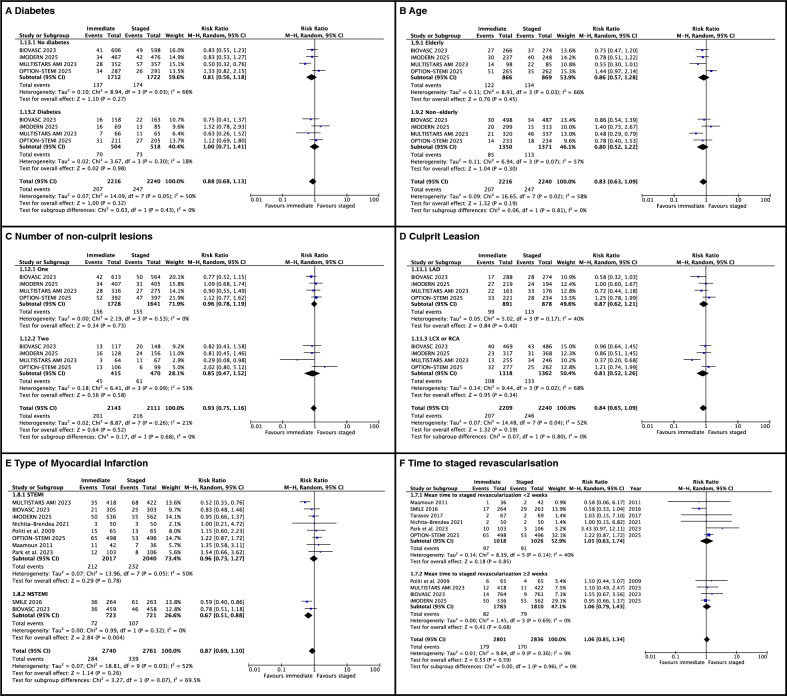
**A–F** Subgroup analyses for the outcome MACE or MACCE at 1 year. Subgroup analyses for the outcome MACE or MACCE (depending on primary outcome of individual trial) are based on **A** the presence of diabetes, **B** age, **C** the number of non-culprit lesions, **D** the localization of culprit lesion (left anterior descending [LAD] versus left circumflex artery [LCX]/right coronary[RCA]), **E** the type of myocardial infarction (ST-elevation [STEMI] versus non-ST-elevation myocardial infarction [NSTEMI]), and **F** the time between the index and staged procedure. Different studies employed varying age thresholds for their subgroup analyses: OPTION-STEMI and iMODERN used < 65 and ≥ 65 years, BIOVASC used < 70 and ≥ 70 years, while MULTISTARS used < 75 and ≥ 75 years

Results of subgroup analyses for MACE or MACCE at 1 year are shown in Fig. 3. With the exception of patients with NSTEMI (Fig. 3E), no subgroup analysis reached statistical significance. In the subgroup of patients presenting with NSTEMI, the risk of developing any MACE or MACCE was significantly lower in the immediate revascularization strategy group (RR 0.67; 95% CI 0.51 to 0.88). However, the subgroup difference was not statistically significant (Chi^2^ = 3.27, p = 0.07, I^2^ = 69.5%).

### Sensitivity analyses

Switching from the random-effects model (RR, 1.10; 95% CI 0.79–1.52) to a fixed-effects model (RR 1.10; 95% CI 0.84–1.43) of the primary outcome all-cause mortality at 1 year did not result in a significant change of the comparison. After the sequential one-by-one exclusion of each individual trial, the pooled summary estimates remained statistically insignificant.

### Meta-regression

In univariate meta-regression analyses, there was no statistically significant association between the risk ratios of the sample size, number of study sites, proportion of patients with chronic hypertension, proportion of male participants, proportion of patients with diabetes, and all-cause mortality (Supplementary Figs. 2, 3, 4, 5, 6, 7).

**Fig. 4:**
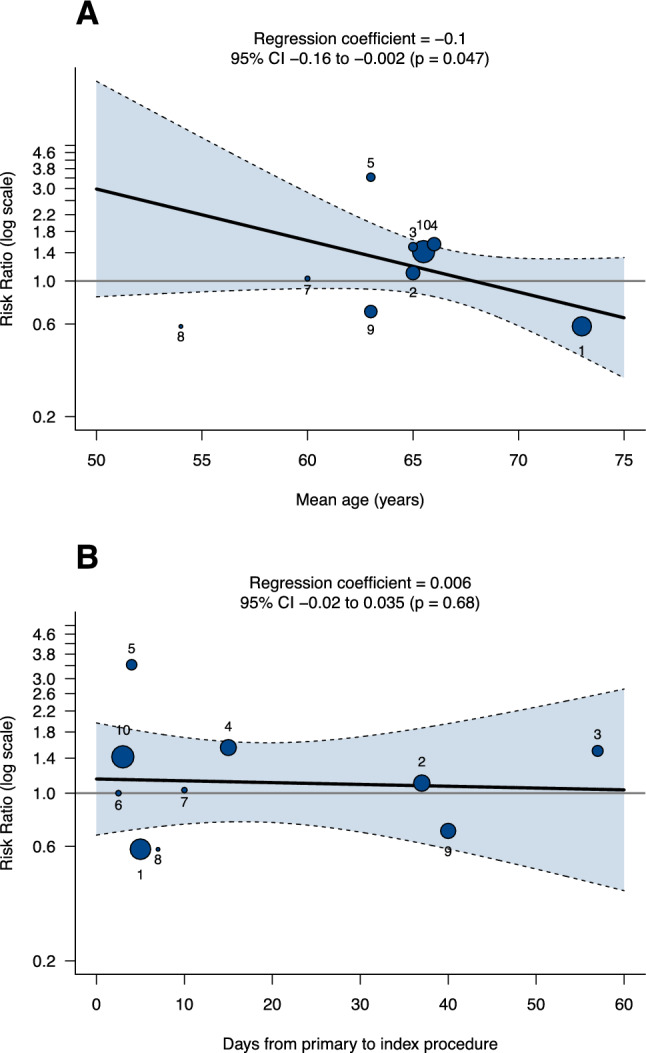
Univariate linear meta-regression analysis between the log-transformed risk ratio of all-cause mortality at 1 year and mean age (**A**) and days from primary procedure to index procedure (**B**). Each study is represented by one circle, with the size of the circle indicating the weight of the study in the analysis. The linear regression line (continuous black line) is presented with 95% confidence intervals (blue area and dashed line). The horizontal grey line indicates a risk ratio of 1 (i.e., no effect). The studies are label as follows: 1 = iMODERN; 2 = OPTION-STEMI; 3 = MULTISTARS AMI; 4 = BIOVASC; 5 = Park et al.; 6 = Nichita-Brendea et al.; 7 = Tarasov et al.; 8 = SMILE; 9 = Maamoun et al.; 10 = Politi et al.

A higher mean patient age was significantly associated with a lower risk ratio favoring the immediate revascularization strategy group (regression coefficient − 0.10; 95% CI − 0.16 to − 0.002; p = 0.047). (Fig. 4A). There was no statistically significant association between the days from index to staged procedure and a greater beneficial effect in the immediate revascularization strategy group (regression coefficient 0.006; 95% CI − 0.02 to 0.035; p = 0.68) (Fig. 4B).

### Risk of bias and certainty of evidence

The most relevant risk of bias arose from the open-label study design, which was unavoidable due to the nature of this clinical situation (Supplementary Table 10). Theoretically, adjudication could have been blinded to minimize the risk of detection bias, but this was not feasible in any of the trials. The primary endpoint of all-cause mortality was unlikely to be affected by this risk of assessment bias. Overall, we rated the certainty of the evidence as moderate, mainly because the 95% confidence interval of the estimated mortality difference is too wide to draw a definitive conclusion on the equivalence between the immediate and staged revascularization strategies (Supplementary Table 11).

## Discussion

In patients presenting with ACS and multivessel disease, it is unclear whether non-culprit lesions should be revascularized immediately or in a staged intervention. This meta-analysis, which pooled data from ten randomized controlled trials with a total of 5651 patients, found similar one-year survival rates between the immediate and staged revascularization strategies.

The 2021 guidelines for coronary revascularization of the American College of Cardiology stated that evidence for immediate complete revascularization of non-culprit lesions in patients with STEMI is lacking [7]. According to more recent 2025 guidelines, immediate complete revascularization is currently recommended for non-culprit lesions in selected patients with ST-elevation myocardial infarction, particularly those with stable hemodynamics and low-complexity coronary anatomy [24]. ESC guidelines for the management of STEMI and NSTEMI recommend complete revascularization of non-culprit lesions but a clear recommendation on the timing of revascularization of non-culprit lesions is lacking [8–10]. All previously mentioned guidelines acknowledged that this topic represents a gap in evidence and encouraged future research on the timing of revascularization of non-culprit lesions. Currently, large randomized-controlled trials are being conducted to determine whether multivessel PCI is superior to culprit-lesion only PCI in patients with NSTEMI and multi-vessel coronary artery disease [25].

The primary endpoint of this meta-analysis was all-cause mortality at 1 year. Mortality was chosen as the primary endpoint, as it is arguably the most relevant and unbiased endpoint for trials investigating the outcomes in patients with acute myocardial infarctions [26]. We found a similar survival following an immediate or staged revascularization strategy. However, the confidence interval for the risk ratio of all-cause mortality at 1 year was too wide (0.79–1.52) to definitively exclude clinically meaningful differences in survival in both directions. Importantly, the observed mortality rates observed in this study are lower than those reported in many observational ACS cohorts, which often reach up to 12% [27]. This discrepancy likely reflects trial selection criteria, including the exclusion of high-risk patients such as those with cardiogenic shock, as well as optimized procedural and medical care within clinical trials.

The individual trials included in this analysis used different study designs. Four trials (OPTION-STEMI, BIOVASC, MULTISTARS AMI, and Park et al.) aimed to show the non-inferiority of immediate complete revascularization, while the trial by Park et al. was prematurely terminated because the production of study-specific stents was halted. The BIOVASC and MULTSTARS AMI trials successfully demonstrated non-inferiority with an absolute non-inferiority margin of 4.0% for MACE and a risk ratio of 1.46 for MACCE, respectively. Even more so, the MULTISTARS AMI trial provided evidence for the superiority of the immediate revascularization strategy with respect to the risk of death from any cause, nonfatal myocardial infarction, stroke, unplanned ischemia-driven revascularization, or hospitalization for heart failure at 1 year. Likewise, the SMILE trial demonstrated the superiority of immediate compared with staged revascularization strategies in terms of major adverse cardiovascular and cerebrovascular events (14% versus 23%). However, the iMODERN trial was designed as a superiority trial, but did not show superiority of immediate (iFR-guided) compared to staged (cardiac stress MRI-guided) revascularization strategies (HR 0.95 [95% CI 0.65–1.40]) with regard to death from any cause, recurrent myocardial infarction, or hospitalization for heart failure at 3 years. In contrast to the previously published studies, the OPTION-STEMI trial did not show non-inferiority of the immediate versus staged revascularization strategy (HR 1.24; 95% CI 0.86–1.79). Politi et al. compared three study arms but found similar outcomes between the immediate and staged revascularization strategy. Furthermore, there are key differences between more recent iMODERN trial and earlier studies. The iMODERN trial incorporated instantaneous wave-free ratio (iFR) guided assessment of non-culprit lesions during STEMI. Physiology-guided assessment using iFR during acute STEMI may be influenced by transient microvascular dysfunction, potentially affecting the accuracy of non-culprit lesion evaluation. In contrast, staged revascularization strategies in some trials incorporated advanced imaging modalities such as stress cardiac magnetic resonance imaging, potentially improving lesion selection. These differences in physiological and imaging-based decision-making may have contributed to divergent findings.

With regard to re-infarction, immediate revascularization almost showed superiority compared with the staged revascularization strategy in the pooled analysis (RR 0.69; 95% CI 0.46–1.03]). Only the OPTION-STEMI, iMODERN, BIOVASC and the MULTISTARS AMI trials provided exact criteria for acute myocardial infarctions. The definitions of myocardial infarctions included the rise of cardiac enzymes accompanied by clinical symptoms and/or ischemic ECG changes. However, this definition may be insufficient in the periprocedural setting and may have led to overestimation of this endpoint in the staged revascularization strategy group. In addition, because of the open-label design, physicians may have been more likely to perform unplanned ischemia-driven revascularization in patients who had not yet undergone the staged procedure than in patients whose lesions had already been stented immediately. Overall, the conflicting findings of a trend towards reduction in myocardial infarction and unplanned revascularization procedures in the immediate revascularization strategy group but similar all-cause mortality highlight the need to focus on mortality as the most robust and objective endpoint available.

Our primary analysis pooled data from patients presenting with acute coronary syndrome with and without ST-elevation. In total, 75% presented with STEMI, 23% presented with NSTEMI, and 2% presented with unstable angina. Our subgroup analysis showed that the risk of MACE or MACCE was significantly lower in the immediate revascularization strategy in the subgroup of patients presenting with NSTEMI, but not in the subgroup of patients presenting with STEMI. Notably, the setting of emergent primary PCI for STEMI differs from the management of NSTEMI, which may allow for delayed PCI depending on the clinical presentation and the patient's individual risk factors. Early, but not emergent, PCI may provide better preconditions for revascularization of non-culprit lesions in the index procedure in patients presenting with NSTEMI. Furthermore, the identification of non-culprit lesions in the setting of STEMI must be based on angiographic severity rather than physiological assessment, which is not recommended in the setting of STEMI by current guidelines [10]. In contrast, functional invasive evaluation of non-culprit lesions during the index procedure may be considered in patients with NSTEMI [10]. The results of the meta-regression indicated a potentially greater survival benefit of the immediate strategy in elderly patients. Although the number of studies included in these meta-regression analyses was small and the age threshold for subgroup analyses varied between studies, older patients with comorbidities may have had higher periprocedural risks and may therefore have benefited more from an immediate revascularization strategy [28]. However, these subgroup and meta-regression findings should be interpreted with caution. Given the limited number of trials, variability in subgroup definitions, and the inherent risk of bias, these results cannot establish a differential treatment effect and should be considered strictly hypothesis-generating. At present, they do not support a strategy selection based solely on age or ACS subtype. Nevertheless, these signals may help inform the design of future adequately powered randomized trials specifically targeting NSTEMI populations and elderly patients to determine whether individualized timing of complete revascularization can improve outcomes.

Potential benefits of an immediate revascularization strategy include a shorter hospital stay, avoiding the increased risk of repeated procedures, improved myocardial perfusion, cost-effectiveness, human resources, and patient comfort. An immediate revascularization strategy may be considered in stable patients and in patients with limited access to healthcare [7]. Moreover, an immediate revascularization strategy may be beneficial in patients with a high risk of subsequent events (e.g., because of high-risk plaque features). Particularly in patients with NSTEMI, the identification of the true culprit may be difficult, and may leave the actual culprit lesions untreated during the index procedure [29–31]. Moreover, non-culprit lesions may also have unstable features that are associated with plaque rupture and the development of re-infarction in the time window between the index and staged procedures [32]. In the BIOVASC trial, the excess of myocardial infarctions in the staged revascularization strategy group was mainly driven by early events that mostly occurred between the index procedure and the planned date for the staged intervention. On the other hand, a staged approach may be beneficial in patients with renal insufficiency (due to a lower dose of contrast used in a single session), in hemodynamically unstable patients, or in those with complex lesions or complex coronary anatomy [7]. In addition, a staged revascularization strategy in patients with STEMI allows physiological assessment (e.g., fractional flow reserve) of non-infarct related arteries, which otherwise must be based on angiography but not on physiological assessment during the index procedure. It should also be noted that nine of the ten included studies excluded patients with cardiogenic shock, making these results inapplicable to this patient population. Finally, from a health-economic perspective, immediate complete revascularization may reduce overall costs by shortening hospital stay and avoiding repeat procedures. Conversely, staged strategies may distribute resource utilization over time but may increase cumulative costs due to additional admissions and procedures. Formal cost-effectiveness analyses are needed to clarify these aspects.

In light of the findings of this meta-analysis, no general recommendation favoring immediate or staged revascularization strategies can be made. Currently, the decision to perform immediate or staged revascularization should be individualized based on the patient's characteristics and clinical situation. Future research should focus on adequately powered randomized controlled trials specifically designed to address the optimal timing of complete revascularization, including stratification by ACS subtype and patient risk profiles. In addition, access to individual patient-level data would allow for more robust subgroup analyses and adjustment for confounding factors that cannot be addressed in trial-level meta-analyses. Finally, the use of standardized endpoint definitions, particularly for composite outcomes such as MACE and MACCE, is essential to improve comparability across studies and strengthen the interpretability of pooled analyses.

Our meta-analysis has several limitations. First, trial-level data was used for this meta-analysis, which is less informative than patient-level data, especially regarding subgroup analyses. Second, although this meta-analysis included all ten available clinical trials, it comprised only 5685 patients, which may be insufficient to detect subtle differences. Third, all included trials had an open-label design inherent to the procedural setting, introducing potential performance bias, particularly for clinician-driven endpoints such as repeat revascularization. This may have influenced the assessment event rates, including re-infarction. While hard endpoints such as all-cause mortality are less susceptible, differences in post-procedural management cannot be excluded. Fourth, the time window from the index procedure to the staged procedure was different among the included trials. Despite this, our meta-regression analysis indicated that variations in staged timing did not appear to significantly influence the primary outcome results. Fifth, there was notable methodological heterogeneity between the included trials. For example, the reliance of iMODERN on iFR-guided lesion assessment, compared with the predominantly angiography-based strategies in earlier trials, introduced design heterogeneity that may have influenced lesion selection and subsequent outcomes. This methodological heterogeneity, together with variability in endpoint definitions across studies, may have influenced secondary outcomes. However, sensitivity analyses confirmed robustness of the primary mortality result. Sixth, important post-procedural factors such as lipid-lowering therapy intensity, achieved LDL-C levels, and adherence to guideline-directed medical therapy were not consistently reported across trials. These factors are known to significantly influence outcomes in ACS and may confound the relationship between revascularization timing and clinical events. Finally, the Cochrane Handbook recommends a minimum of 10 studies per covariate examined in meta-regression [33]. While we reached the minimum number of recommended studies, a higher number of studies would have increased the validity of the analysis. However, it has also been argued that fewer observations are sufficient for univariate linear meta-regression analyses [34, 35].

## Conclusions

In patients presenting with ACS, predominantly with STEMI, and multivessel coronary disease, all-cause mortality and cardiovascular deaths at 1 year were similar between the immediate and staged revascularization strategy groups. The available effect estimates do not exclude the possibility of clinically meaningful benefit or harm from immediate revascularization of nonculprit lesions. Furthermore, the results in patients with NSTEMI should be considered exploratory due to the limited representation of this subgroup (Fig. 3E).

## Supplementary Information

Below is the link to the electronic supplementary material.

Supplementary file1 (DOCX 596 KB)

## Data Availability

All data used in this meta-analysis is publicly available. Specific data can be made available upon request by the corresponding author.
