# Supplementary material for: Immediate versus staged revascularization in acute coronary syndrome and multivessel disease: a meta-analysis and meta-regression of RCTs
Source: Intern Emerg Med. 2026 May 12;21(4):1469–80. doi: 10.1007/s11739-026-04369-z (PMC13263209; doi:10.1007/s11739-026-04369-z)
Supplement: Supplementary file 1 — Supplementary file1 (DOCX 596 KB) [file 11739_2026_4369_MOESM1_ESM.docx]

**Supplementary Material**

**Immediate versus staged revascularization in acute coronary syndrome and multivessel disease: a meta-analysis and meta-regression of RCTs**

**Brief Title: Meta-analysis of immediate versus staged revascularization**

Felix Bergmann, MD, PhD^a^ †, Anselm Jorda, MD^a^ †, Theresa Pecho^a^, Lukas Stoiber^a^, Georg Gelbenegger, MD PhD^a^, Lena Pracher, MD^a^, Amelie Leutzendorff, MD^a^, Gregor Heitzinger, MD PhD^b^, Jolanta M. Siller-Matula, MD, PhD^b,c^, Christian Schörgenhofer, MD, PhD^a^, Markus Zeitlinger, MD^a^, Irene Lang, MD^b^, Valentin al Jalali, MD, PhD^a*^

^a^Department of Clinical Pharmacology, Medical University of Vienna, Vienna, Austria

^b^Department of Medicine II, Division of Cardiology, Medical University of Vienna, Vienna, Austria

^c^Department of Experimental and Clinical Pharmacology, Centre for Preclinical Research and Technology (CEPT), Medical University of Warsaw, Warsaw, Poland

**† Contributed equally**

***Corresponding Author (Address for correspondence)**

Valentin al Jalali

Department of Clinical Pharmacology, Medical University of Vienna

Waehringer Guertel 18-20, 1090 Vienna, Austria

valentin.aljalali@meduniwien.ac.at

Tel: +43 1 40400 29810

**Funding**

This research did not receive external funding.

**Disclosures**

The authors have nothing to declare.

**Table of content**

[Supplementary Tables 3](#_Toc214537007)

[Supplementary Table 1: Systematic search strategy on Pubmed (MEDLINE), Embase, Web of Science, and Cochrane Library (Date of search November 12, 2025) 3](#_Toc214537008)

[Supplementary Table 2: Exact definition of major adverse cardiovascular events (MACE) and major adverse cardiovascular and cerebrovascular events (MACCE) 4](#_Toc214537009)

[Supplementary Table 3: Definitions of myocardial infarctions 5](#_Toc214537010)

[Supplementary Table 4: Inclusion and exclusion criteria 8](#_Toc214537011)

[Supplementary Table 5: Baseline characteristics of study participants 10](#_Toc214537012)

[Supplementary Table 6: Location of culprit lesions 11](#_Toc214537013)

[Supplementary Table 7: Classification of lesion complexity according to the According to the American College of Cardiology/American Heart Association criteria. 12](#_Toc214537014)

[Supplementary Table 8: Medical treatment at discharge 13](#_Toc214537015)

[Supplementary Table 9: Procedural characteristics 14](#_Toc214537016)

[Supplementary Table 10: Risk of Bias Assessment according to the Revised Cochrane risk-of-bias tool for randomized trials (RoB 2) 15](#_Toc214537017)

[Supplementary Table 11: Assessment of level of certainty of evidence according to GRADE recommendations 16](#_Toc214537018)

[Supplementary Figures 17](#_Toc214537019)

[Supplementary Figure 1: PRISMA Flowchart of identified records 17](#_Toc214537020)

[Supplementary Figure 2: Univariate linear meta-regression analysis between the log- transformed risk ratio of all-cause mortality at 1 year and total sample size in the intention-to-treat population of each study. 18](#_Toc214537021)

[Supplementary Figure 3: Univariate linear meta-regression analysis between the log- transformed risk ratio of all-cause mortality at 1 year and the number of study sites of each study. 19](#_Toc214537022)

[Supplementary Figure 4: Univariate linear meta-regression analysis between the log- transformed risk ratio of all-cause mortality at 1 year and the percentage of patients with chronic hypertension. 20](#_Toc214537023)

[Supplementary Figure 5: Univariate linear meta-regression analysis between the log- transformed risk ratio of all-cause mortality at 1 year and the proportion of male participants (%) of each study. 21](#_Toc214537024)

[Supplementary Figure 6: Univariate linear meta-regression analysis between the log- transformed risk ratio of all-cause mortality at 1 year and the proportion of patients with diabetes (%) of each study. 22](#_Toc214537025)

[Supplementary Figure 7: Univariate linear meta-regression analysis between the log- transformed risk ratio of all-cause mortality at 1 year and the event rate of all-cause mortality within each study. 23](#_Toc214537026)

# Supplementary Tables

## Supplementary Table 1: Systematic search strategy on Pubmed (MEDLINE), Embase, Web of Science, and Cochrane Library (Date of search November 12, 2025)

| **Pubmed (November 12, 2025)** | **N=** |
| --- | --- |
| ((immediate*[Title]) OR (staged*[Title])OR (step*[Title]) OR (delayed*[Title]) OR (deferred*[Title])) AND ((coronar*[Title]) OR (infarction*[Title]) OR (revascul*[Title]) OR (myocardial*[Title]) OR (elevation*[Title]) OR (STEMI*[Title]) OR (NSTEMI*[Title])) | **3,495** |
| ((immediate*[Title]) OR (staged*[Title])OR (step*[Title]) OR (delayed*[Title]) OR (deferred*[Title])) AND ((coronar*[Title]) OR (infarction*[Title]) OR (revascul*[Title]) OR (myocardial*[Title]) OR (elevation*[Title]) OR (STEMI*[Title]) OR (NSTEMI*[Title])) AND (randomized controlled trial[Filter]) | **222** |
| **Embase** | |
| (immediate:ti OR staged:ti OR step:ti OR delayed:ti OR deferred:ti) AND (coronary:ti OR infarction:ti OR revascul*:ti OR myocardial:ti OR elevation:ti OR stemi:ti OR nstemi:ti) | **4,289** |
| (immediate:ti OR staged:ti OR step:ti OR delayed:ti OR deferred:ti) AND (coronary:ti OR infarction:ti OR revascul*:ti OR myocardial:ti OR elevation:ti OR stemi:ti OR nstemi:ti) AND 'trial':ab,ti | **309** |
| **Web of Science** | |
| (TI=(immediate) OR TI=(staged) OR TI=(delayed) OR TI=(deferred)) AND (TI=(coronary) OR TI=(infarction) OR TI=(revascul*) OR TI=(myocardial) OR TI=(elevation) OR TI=(stemi) OR TI=(nstemi)) | **7356** |
| (TI=(immediate) OR TI=(staged) OR TI=(delayed) OR TI=(deferred)) AND (TI=(coronary) OR TI=(infarction) OR TI=(revascul*) OR TI=(myocardial) OR TI=(elevation) OR TI=(stemi) OR TI=(nstemi))AND (TI=(trial) OR AB=(trial)) | **544** |
| **Cochrane Library** | |
| ((immediate):ti OR (staged):ti OR (delayed):ti OR (deferred):ti) AND ((coronary):ti OR (revascul*):ti OR (myocardial):ti OR (elevation):ti OR (stemi):ti) OR (nstemi):ti) | **396** |
| ((stroke):ti OR (cerebrovascular):ti OR (apoplex):ti OR (accident):ti OR (event):ti OR (brain):ti OR (ischemic):ti OR (ischemia):ti OR (insult):ti,ab,kw) AND ((thrombectomy):ti OR (endovascular):ti OR (mechanical):ti OR (invasive):ti OR (extraction):ti,ab,kw) AND ((lysis):ti OR (thrombolysis):ti OR (tpa):ti OR ('t pa'):ti OR (plasmin):ti OR (alteplase):ti OR (thrombolytic):ti) AND ENGLISH | **200** |

## Supplementary Table 2: Exact definition of major adverse cardiovascular events (MACE) and major adverse cardiovascular and cerebrovascular events (MACCE)

| **Study** | Text | Death | Myocaridal infarction | Revascularization | Stroke | Rehospitalization |
| --- | --- | --- | --- | --- | --- | --- |
| BIOVASC,  2023 | All-cause death, myocardial infarction, unplanned ischemia-driven revascularization, or cerebrovascular events | + | + | + | + | - |
| Politi et al,  2009 | Cardiac or non-cardiac death, in-hospital death, re-infarction, re-hospitalization for ACS and repeat coronary revascularization | + | + | + | - | + |
| SMILE,  2016 | Cardiac death, death, reinfarction, rehospitalization for unstable angina, repeat coronary revascularization (target vessel revascularization), and stroke | + | + | + | + | + |
| Park et al.,  2023 | Composite of total death, recurrent MI, repeat revascularization, and any individual component of MACE | + | + | + | - | - |
| MULTISTARS AMI,  2023 | Composite of death from any cause, nonfatal myocardial infarction, stroke, unplanned ischemia-driven revascularization, or hospitalization for heart failure | + | + | + | + | + |
| Nichita-Brendea et al., 2021 | Composite of all-cause mortality, new myocardial infarction, stroke, and symptom-driven revascularization at one year | + | + | + | + | - |
| Tarasov et al., 2017 | Combined endpoint of death, myocardial infarction, and target vessel revascularization | + | + | + | - | - |
| Maamoun et al., 2010 | Including death (cardiac or non-cardiac), recurrent MI, rehospitalization because of recurrent angina, target vessel revascularization and cerebrovascular accidents, defined as any neurologic event whether hemorrhagic or non-hemorrhagic stroke | + | + | + | + | + |
| OPTION-STEMI  2025 | Death from any cause, nonfatal myocardial infarction, or all unplanned repeat revascularization | + | + | + | - | - |
| iMODERN  2025 | Composite of death from any cause, recurrent MI, or hospitalization for  heart failure at 3 yr | + | + | - | - | + |

## Supplementary Table 3: Definitions of myocardial infarctions

| **Definition of myocardial infarctions in the BIOVASC trial** |
| --- |
| Any of the following criteria meets the diagnosis of myocardial infarction:  **1. Spontaneous myocardial infarction**  a) In patients with initial normal baseline cardiac troponin (cTn) values Clinical evidence of acute myocardial ischaemia and with detection of a rise and/or fall of cTn values with at least one value above the 99th percentile upper reference limit (URL) and at least one of the following:  o Symptoms of myocardial ischaemia  o New ischaemic ECG changes  o Development of pathological Q waves  o Imaging evidence of new loss of viable myocardium or new regional wall motion abnormality in a pattern consistent with an ischaemic aetiology  o Identification of a coronary thrombus by angiography or autopsy  b) In patients whose cardiac troponin values are already elevated or were recently elevated New ischemic symptoms of the duration of at least 20 minutes and new ischaemic ECG changes are required. These ECG changes must be distinct from the original MI and not due to the usual ECG evolution of this event.  **2. Patients who suffer cardiac death**, with symptoms suggestive of myocardial ischaemia accompanied by presumed new ischaemic ECG changes or ventricular fibrillation but die before blood samples for biomarkers can be obtained, or before increases in cardiac biomarkers can be identified, or MI is detected by autopsy examination.  **3. Procedural related myocardial infarction**  Procedural related myocardial infarction (within 48 Hours) for Percutaneous Coronary Intervention and Coronary Artery Bypass Grafting is defined:  a) In patients with initial normal baseline cardiac troponin (cTn) values. Absolute rise in cardiac troponin (from baseline) ≥35 times the 99th percentile URL or CK- MB >5 times the 99th percentile URL and with at least one of the following criteria:  o Symptoms of myocardial ischaemia  o New ischaemic ECG changes  o Angiographic evidences of major procedural complications  o Imaging evidence of new loss of viable myocardium or new regional wall motion abnormality consistent with an ischaemic aetiology  b) In patients whose cardiac troponin values are already elevated or were recently elevated (i.e.,suspected new myocardial infarction occurs within 1 week of the last troponin measurement), new ischemic symptoms of at least 20 minutes and either new ST segment elevation of at least 1mm in 2 adjacent limb leads or 2 mm in 2 adjacent precordial leads are required. These ECG changes must be distinct from the original myocardial infarction and not due to the usual ECG evolution of this event.  **4. Stent thrombosis** associated with myocardial infarction when detected by coronary angiography or autopsy |
| **Definition of myocardial infarctions in the MULTISTARS AMI trial** |
| Myocardial infarction will be defined based on the third universal definition:  The term acute MI should be used when there is evidence of myocardial necrosis in a clinical setting consistent with myocardial ischemia. Under these conditions, any one of the following criteria met the diagnosis for MI:  **1. Detection of rise and/or fall of cardiac biomarkers** (preferably cardiac troponin (cTn) with at least one value above the 99th percentile of the upper reference limit (URL) and with at least one of the following: a. Symptoms of ischemia;  b. New significant ST segment alterations, T-wave changes, or new left bundle branch block (LBBB); c. Development of pathological Q waves in the ECG; d. Imaging evidence of new loss of viable myocardium;  e. Identification of an intracoronary thrombus by angiography.  **2. Cardiac death** with symptoms suggestive of myocardial ischemia and presumed new ischemic ECG changes or new LBBB, but death occurred before cardiac biomarkers were obtained, or before cardiac biomarker values would be increased.  **3. PCI-related MI** (MI type4a): Elevation of cardiac troponin (cTn) values >5x99th percentile URL occurring within 48 hours of the procedure in patients with normal baseline values (≤99th percentile URL), or a rise of cTn values >20 percent if the baseline values were elevated or were stable or falling. In addition, either new or aggravating prolonged (>20 min) symptoms suggestive of myocardial ischemia, or new persistent ischemic ST segment changes or new pathological Q waves, or angiographic evidence of a flow-limiting complication such as persistent occlusion or persistent slow-flow, no-reflow, or embolization, and/or angiographic evidence of persistent loss of patency of a major (≥ 2.0 mm) side branch, or imaging evidence of new loss of viable myocardium or new regional wall motion abnormality were required. A rise of cardiac enzymes post  procedure represents an inherent condition in the course of an acute MI treated with primary PCI. Therefore, in case of new persistent ischemic ST segment changes or new/aggravating prolonged (>20 min) symptoms suggestive of myocardial ischemia, within 48 hours of the procedure, a coronary angiogram was recommended to confirm or exclude peri-procedural MI. Troponin I or T were used first, if not available, the second option was creatine kinase- myocardial band (CK-MB), and if CK-MB was not available, total CK was used.  **4. Stent thrombosis** associated with MI when detected by coronary angiography or autopsy in the setting of myocardial ischemia and with a rise and/or fall of cardiac biomarker values with at least one value above the 99th percentile.  **5. For coronary artery bypass grafting** (CABG), in patients with normal baseline cTn values (≤99th percentile URL), procedure related MI was arbitrarily defined by elevations of >10 x 99th percentile plus either new pathological Q waves or new LBBB, or angiographic documented new graft or native coronary artery occlusion, or imaging evidence of new loss of viable myocardium.  **6. Pathological findings** of an acute or a recent MI. |
| **Definition of myocardial infarctions in the iMODERN trial** |
| **Definition of spontaneous myocardial infarction**  Spontaneous myocardial infarction may be considered after the first 48 hours after randomization or  secondary PCI and is defined as one of the following:  • Typical rise and/or fall of biochemical markers of myocardial necrosis with at least one of the  following:  - Symptoms of ischemia  - New ECG changes suggestive of ischemia (ST-elevation, ST-depression or T-wave  abnormalities)  - Development of new pathologic Q-waves on the ECG  • Development of new pathologic Q-waves on follow-up ECG in the absence of cardiac  biomarker assessment during the acute event.  • Pathological findings of an acute myocardial infarction during autopsy  **Definition of periprocedural myocardial infarction**  Periprocedural myocardial infarction may be considered within the first 48 hours after PCI and its  definition depends on the setting.  During acute myocardial infarction:  Periprocedural myocardial infarction in the setting of evolving myocardial infarction can only be  diagnosed after a peak CK-MB or Troponin has been reached and is defined as one of the following:  • Postprocedural elevation in CK-MB > 3 times upper limit of normal or Troponin >5 times the 99  percentile, if the biomarkers had returned to below the upper limit of normal prior to PCI.  • A rise of >50% above the previous peak level in CK-MB or Troponin, if the biomarkers were  still above the upper limit of normal prior to PCI.  AND in combination with at least one of the following:  • New pathological Q waves in at least 2 contiguous leads or new persistent non-rate  dependent left bundle branch block.  • Symptoms of ischaemia along with new ECG changes indicative of ischemia.  • Angiographic documentation of new coronary artery (side branch) occlusion or dissection.  During elective PCI:  Elective PCI may occur as treatment of nonculprit lesions in the deferred cardiac MRI group or as a  result of ischemic symptoms during follow-up. In the setting of elective PCI, periprocedural related  myocardial infarction is defined as one of the following:  • Postprocedural elevation in CK-MB>3 times upper limit of normal or Troponin >5 times the 99  percentile, if the biomarkers were not elevated prior to PCI.  • A rise of >50% above the previous level of CK-MB or Troponin, if CK-MB or Troponin were  elevated prior to PCI but without a rise and/or fall (e.g. renal insufficiency). |
| Definition of myocardial infarctions in the OPTION-STEMI trial |
| Myocardial Infarction (MI): In accordance with the Fourth Universal Definition of Myocardial  Infarction, MI will be classified to spontaneous or procedure-related MI.  **Spontaneous MI:** MI was defined as acute myocardial injury with clinical evidence of acute myocardial  ischemia. Under these conditions, any one of the following criteria will meet the diagnosis of  spontaneous MI.  1) Detection of a rise and/or fall of cardiac troponin (cTn) level with at least one value above the 99th  percentile of the upper reference limit (URL) and at least one of the following:  • Symptoms of MI  • New ischemic electrocardiographic changes: new or presumed new significant ST-T wave (ST-  T) changes or new left bundle branch block  • Development of pathological Q waves  • Imaging evidence of new loss of viable myocardium or new regional wall motion abnormality  in a pattern consistent with an ischemic etiology  • Identification of a coronary thrombus by angiography or autopsy  2) Cardiac death in patients with symptoms suggestive of MI and presumed new ischemic  electrocardiographic changes before the cTn value become available or if the cTn value is abnormal.  3) Stent thrombosis associated with MI when detected by coronary angiography or autopsy in the setting  of myocardial ischemia associated with a rise and/or fall in cTn value with at least one value above  12the 99th percentile of the URL  **Procedure-related MI:**  In patients with normal baseline cTn values, the definition is based by an elevation of cTn values >5  times the 99th percentile URL within 48 hours of the procedure. In patients with elevated preprocedure  cTn in whom the cTn level are stable (≤20% variation) or falling, the postprocedure cTn must rise by  >20% within 48 hours of the procedure. However, the absolute postprocedural value must still be at  least 5 times the 99th percentile URL. In addition, 1 of the following elements is required:  • New ischemic electrocardiographic changes  • Development of new pathological Q waves  • Imaging evidence of new loss of viable myocardium or new regional wall motion abnormality  in a pattern consistent with an ischemic etiology  • Angiographic findings consistent with a procedural low-limiting complication such as  coronary dissection, occlusion of a major epicardial artery or a side branch occlusion/thrombus,  disruption of collateral flow, or distal embolization. |

## Supplementary Table 4: Inclusion and exclusion criteria

|  | **BIOVASC** | **Politi et al.** | **SMILE** | **Park et al.** | **MULTISTARS AMI** | **Nichita-Brendea et al.** | **Tarasov et al.** | **Maamoun et al.** | **OPTION-STEMI** | **iMODERN** |
| --- | --- | --- | --- | --- | --- | --- | --- | --- | --- | --- |
| **Inclusion criteria** | | | | | | | | | | |
| **Age** | • ≥18 y and ≤85 y | na | • > 18 y | • ≥18y | • ≥18y | • ≥18y to 90y | • ≥18y | na | ≥19y | ≥18y |
| **Clinical presentation** | • ACS (Unstable angina, NSTEMI, or STEMI) | • Presence of prolonged (> 30 min) chest pain • Started less than 12 h before hospital arrival and ST elevation of at least 1 mm in two or more contiguous limb electrocardiographic leads or 2 mm in precordial leads | • NSTEMI • presenting with multivessel disease | •STEMI | • STEMI | • STEMI. PCI for STEMI should be primary PCI in the first 12 hours after symptom onset. | • STEMI | • STEMI | • STEMI  • ST-segment elevation ≥0.1 mV in ≥2 contiguous leads or  • New onset New onset left bundle branch block | • STEMI and successful primary PCI within 12 hours from onset of  Symptoms. |
| **Other** | • The patient is an acceptable candidate for treatment with a DES • The patient is willing and able to cooperate with study procedures • The subject or legal representative has been informed of about the study | na | • Glomerular filtration rate >60 ml/min  • Planned early invasive strategy • Signed informed consent | • >2 significant target lesions in different target native coronary arteries requiring PCI within 72 hours • Target lesion is amenable | • Suitable for PCI  • Identifiable culprit lesion  • Coronary anatomy suitable for complete PCI  • Coronary stenosis ≥ 70 in a vessel with a lumen diameter ≥ 2.25 - ≤ 5.75 mm other than the culprit artery  • Thrombolysis in Myocardial Infarction (TIMI) Flow grade 2 or 3 after revascularization of the culprit artery  •Hemodynamically stable | •MVD defined as at least 1 additional non-infarct related coronary artery lesion that is at least 2 mm in diameter that has not been stented as part of the primary PCI and is amenable to successful treatment with PCI and has: ≥75% stenosis or ≥50% diameter stenosis with fractional flow reserve ≤0.80  • interventional cardiologist had to regard PCI as a valid option to treat all significant stenoses | • Multivessel native coronary arteries significant stenosis and primary PCI ≤12 h from STEMI manifestations and ST-segment elevation ≥1 mm in ≥2 limb electrocardiographic leads or precordial leads ≥2 mm ST-segment elevation  • Diameter of coronary artery ≥2.5 mm  • ≥30 minutes chest pain. | na | • Primary PCI within 12 hours after symptom onset  • Multivessel disease: non-IRA vessel ≥2.5 mm and ≥50% stenosis by visual estimation  • Patient’s or guardian’s consent after understanding the study | • One or more other, nonculprit coronary artery lesions of >50% stenosis and feasible to be  revascularized with PCI (i.e., minimal diameter 2mm) |
| **Exclusion criteria** | | | | | | | | | | |
| **Cardiogenic shock** | • Cardiogenic shock | • Cardiogenic shock | • Cardiogenic shock | • Cardiogenic shock | • Cardiogenic shock | • Cardiogenic shock  • Cardiac arrest | • Cardiogenic shock | na | • Cardiogenic shock at initial presentation or after IRA treatment | • Hemodynamic instability / cardiogenic shock (Killip class ≥ III) |
| **Coronary artery features** | • Single coronary artery disease • If it is unclear which lesion is the culprit lesion  • Presence of a chronic total occlusion  • Previous CABG | • Left main coronary disease (>50% diameter stenosis) • Previous CABG | • Chronic total occlusion • Previous CABG | na | • Need for emergency CABG.  • Previous CABG.  • Coronary artery dissection  • Stent thrombosis  • In-stent restenosis  • Chronic total occlusion of a major coronary artery  • Left main disease (≥ 50% stenosis) or left main equivalent (ostial left  anterior descending and ostial circumflex stenosis ≥ 70%) | • Prior CABG surgery | • Significant left main stenosis (≥50%)  • No MVCAD | na | • Unprotected left main disease ≥50% stenosis  • TIMI flow at non-IRA ≤2  • Non-IRA lesion unsuitable for PCI (operator judgement)  • Chronic total occlusion at non-IRA | • History of ST-elevation myocardial infarction  • Coronary artery bypass graft  • Chronic total  occlusion  • Left main stenosis > 50%  • Residual nonculprit lesion in culprit vessel  • Complex nonculprit target lesions |
| **Other** | • Age <18 y and >85 y  • Patients who cannot give informed consent • Life expectancy < 1 y  • Absolute contraindications or allergy that cannot be premedicated to iodinated contrast or to any of the study medications, including both aspirin and P2Y12 inhibitors  • Enrolment in another study with another investigational device or drug trial that has not reached the primary end point. • PCI in the previous 30 d  • Women of childbearing potential who do not have a negative pregnancy test result within 7 d before the procedure and women who are breastfeeding  • Planned surgery within 6 m after PCI unless dual antiplatelet therapy is maintained throughout the peri-surgical peridod | •Severe valvular heart disease and unsuccessful procedures | • SYNTAX score >32  • Candidate for bypass surgery • Severe valvular heart disease | • Adverse reactions to heparin, aspirin, clopidogrel, ticlopidine, everolismus, and contrast agent  • Systemic (iv) everolimus use within 12 months,  • Pregnancy • History of bleeding diathesis or known coagulopathy • Planned elective surgical procedure that would necessitate thienopyridine interruption during the first 6 months • Life expectancy <1 year • Actively participating in another drug or device investigational study,  • LVEF < 25% | • Prolonged resuscitation > 10 min.  • Allergy to everolimus or any stent material  • Planned hybrid revascularization  • Mechanical complication of acute MI  • Pre-existing severe renal failure (estimated glomerular filtration rate <30 mL/min)  • Planned coronary, cerebrovascular, or peripheral arterial revascularization  • Planned cardiac or major surgery  • Any contraindications for DAPT with aspirin and a P2Y12 inhibitors  • Pregnancy  • Participation in another clinical study with an investigational product  • Life expectancy < 1 year. | • Rescue PCI for failed fibrinolysis or a combination strategy where PCI is performed routinely 3–12 h after fibrinolysis.  • Planned surgical revascularization  • Non-cardiovascular known co-morbidity reducing life expectancy to <2 years.  • Any factor precluding one-year follow-up.  • A different operator from the previously designated.  • Unable to provide consent for any other reason.  • Impaired renal function (GFR <60 mL/min/1.73 m2). | • Contraindication to use heparin, aspirin, clopidogrel, ticagrelor, zotarolimus | na | • Severe procedural complications (e.g., no-reflow, perforation)  • History of anaphylaxis to contrast agent  • Pregnancy or lactation  • Life expectancy <1 year  • Severe valvular disease  • History of CABG, or planned CABG  • Fibrinolysis before admission  • Severe asthma  • Patient refusal to participate | • Respiratory failure  • GFR < 30 mL/min  • Contraindications to stress cardiac MRI  • Life expectancy < 12 months (non-cardiovascular)  • Inability or refusal to provide informed consent  • Pregnancy |

## Supplementary Table 5: Baseline characteristics of study participants

|  | BIOVASC | | Politi et al. | | SMILE | | Park et al. | | MULTISTARS AMI | | Nichita-Brendea et al. | | Tarasov et al. | | Maamoun et al. | | OPTION-STEMI | | IMODERN | |
| --- | --- | --- | --- | --- | --- | --- | --- | --- | --- | --- | --- | --- | --- | --- | --- | --- | --- | --- | --- | --- |
|  | Immediate | Staged | Immediate | Staged | Immediate | Staged | Immediate | Staged | Immediate | Staged | Immediate | Staged | Immediate | Staged | Immediate | Staged | Immediate | Staged | Immediate | Staged |
| N of patients | 764 | 761 | 65 | 65 | 264 | 263 | 103 | 106 | 418 | 422 | 50 | 50 | 67 | 69 | 42 | 36 | 498 | 496 | 556 | 587 |
| Age (years) | 66 (57-73) | 65 (59-73) | 65 ± 12 | 64 ± 11 | 72 (61-78) | 73 (62-78) | 63 ± 10 | 62 ± 11 | 66 (58-74) | 64 (55-73) | na | na | 59 ± 10 | 59 ± 11 | 54±10 | 52±7 | 66·0 (57·0–76·0) | 65·0 (58·0–76·0) | 62.7±11.3 | 62.7±11.0 |
| Men (%) | 598 (78%) | 589 (77%) | 50 (77%) | 52 (80%) | 207 (78%) | 209 (79%) | 82 (80%) | 88 (83%) | 321 (77%) | 341 (81%) | 37 (74) | 36 (72) | 48 (72%) | 43 (62%) | 40 (95%) | 32 (89%) | 396 (80%) | 393 (79%) | 77.8% | 77.6% |
| Women (%) | 166 (22%) | 172 (23%) | 15 (23%) | 13 (20%) | 57 (22%) | 54 (21%) | 21 (20%) | 18 (17%) | 97 (23%) | 81 (19%) | 13 (26) | 14 (28) | 29 (28%) | 26 (38%) | 2 (5%) | 4 (11%) | 102 (20%) | 103 (21%) | 22.2% | 22.4% |
| BMI (kg/m^2) | 27 (25-30) | 27 (25-30) | na | na | na | na | 25 ± 4.2 | 24 ± 2.7 | na | na | na | na | na | na | 21.6±2.9 | 20.8±3.1 | 24·0 (22·1–26·0) | 24·2 (22·0–26·3) | 27.4±4.5 (n = 468) | 27.2±4.3 (n = 528) |
| Diabetes (%) | 158 (21%) | 163 (21%) | 9 (14%) | 12 (18%) | 98 (37%) | 104 (40%) | 44 (43%) | 40 (38%) | 66 (16%) | 65 (15%) | 12 (24%) | 11 (22%) | 16 (24%) | 14 (20%) | 17 (41%) | 20 (56%) | 211 (42%) | 205 (41%) | 73/554 (13.2%) | 90/585 (15.4%) |
| Hypertension (%) | 423 (55%) | 395 (52%) | 32 (49%) | 42 (65%) | 193 (73%) | 174 (66%) | 56 (54%) | 48 (45%) | 228 (55%) | 212 (50%) | 20 (40%) | 24 (48%) | 64 (94%) | 61 (88.4%) | 16 (38%) | 12 (33%) | 245 (49%) | 253 (51%) | 207/554 (37.4%) | 228/585 (39.0%) |
| Systolic blood pressure (mmHg) | 125 (110-140) | 125 (110-140) | 136 ± 24 | 136 ± 31 | 130 (120-140) | 125 (115-135) | 134 ± 27 | 128 ± 24 | na | na | na | na | na | na | na | na | 120 (110–140) | 120 (110–140) | na | na |
| Diastolic blood pressure (mmHg) | 72 (63-81) | 71 (62-80) | na | na | na | na | 80 ± 18 | 79 ± 15 | na | na | na | Na | na | na | na | na | 80 (64–80) | 80 (61–80) | na | na |
| Heart rate (1/min) | na | na | na | na | 80 (67-90) | 78 (65-88) | 78 ± 19 | 76 ± 17 | na | na | na | na | na | na | na | na | 77 (65–90) | 80 (67–90) | na | na |
| Current smokers (%) | 254 (33%) | 240 (32%) | na | na | 120 (45%) | 107 (41%) | 38 (37%) | 44 (42%) | 140 (34%) | 149 (35%) | na | na | na | na | na | na | 205 (41%) | 202 (41%) | na | na |
| Former smokers (%) | 145 (19%) | 148 (19%) | na | na | na | na | 16 (16%) | 12 (11%) | 78 (19%) | 57 (14%) | na | na | na | na | na | na | 97 (19%) | 85 (17%) | na | na |
| Previous myocardial infarction (%) | 69 (9%) | 89 (12%) | na | na | 71 (27%) | 62 (24%) | 1 (1%) | 1 (1%) | 28 (7%) | 20 (5%) | na | na | 10 (14.9%) | 4 (5.8%) | na | na | 40 (8%) | 36 (7%) | na | na |
| Renal insufficiency (%) | 41 (5%) | 37 (5%) | 17 (26%) | 16 (25%) | na | na | na | na | na | na | na | na | na | na | na | na | 37 (7%) | 34 (7%) | 8/554 (1.4%) | 10/585 (1.7%) |
| Dyslipidaemia (%) | 385 (51%) | 399 (53%) | na | na | 152 (58%) | 143 (54%) | 38 (37%) | 41 (39%) | 112 (27%) | 114 (27%) | na | na | na | na | 24 (57%) | 16 (44%) | 295 (59%) | 280 (56%) | 151/554 (27.3%) | 180/585 (30.8%) |
| Previous PCI (%) | 83 (11%) | 121 (16%) | na | na | 41 (16%) | 44 (17%) | 2 (2%) | 0 (0%) | 33 (8%) | 23 (5%) | na | na | na | na | na | na | 49 (10%) | 50 (10% | na | na |
| LVEF before PCI (%) | na | na | 45 ± 10 | 46 ± 8.6 | 50 (40-55) | 50 (40-55) | 51 ± 11 | 52 ± 11 | na | na | 42 (40-44) | 45(43-47) | na | na | 45±7 | 46±9 | 50·3 | 49·7 | na | na |
| Door to balloon time (min) | na | na | 60 ± 23 | 66 ± 19 | na | na | 86 ± 84 | 83 ± 89 | na | na | na | na | na | na | na | na | 69 (55–89) | 69 (56–90) | na | na |
| Length of hospital stay (days) | 3 (2-5) | 4 (3-6) | 4.8 ± 2.6 | 5.4 ± 3.1 | na | na | 11 ± 26 | 9.0 ± 10 | 4 (3-6) | 5 (4-7) | na | na | na | na | na | na | 4 (3–6) | 5 (4–8) | na | na |

Data are expressed as median (interquartile range), mean ± standard deviation, or number (%).

## Supplementary Table 6: Location of culprit lesions

|  | BIOVASC | | Politi et al. | | SMILE | | Park et al. | | MULTISTARS AMI | | Nichita-Brendea et al. | | Tarasov et al. | | Maamoun et al. | | OPTION-STEMI | | iMODERN | | Total | |
| --- | --- | --- | --- | --- | --- | --- | --- | --- | --- | --- | --- | --- | --- | --- | --- | --- | --- | --- | --- | --- | --- | --- |
|  | Immediate | Staged | Immediate | Staged | Immediate | Staged | Immediate | Staged | Immediate | Staged | na | na | na | na | na | na | Immediate | Staged | Immediate | Staged | Immediate | Staged |
| N of culprit lesions | 757 | 760 | na | na | na | na | 104 | 108 | 418 | 423 | na | na | na | na | na | na | 498 | 496 | 556 | 587 | 2333 | 2374 |
| Left main coronary artery | 3 (0%) | 5 (1%) | na | na | na | na | 1 (1%) | 1 (1%) | 0 (0%) | 1 (0%) | na | na | na | na | na | na | na | na | na | na | 4 (0%) | 7 (0%) |
| Left anterior descending artery | 285 (38%) | 269 (35%) | na | na | na | na | 45 (43%) | 48 (44%) | 163 (39%) | 176 (42%) | na | na | na | na | na | na | 221 (44%) | 234 (47%) | 233 (41.9%) | 218 (37.1%) | 947 (41%) | 945 (40%) |
| Circumflex artery | 209 (28%) | 203 (27%) | na | na | na | na | 12 (12%) | 17 (16%) | 67 (16%) | 77 (18%) | na | na | na | na | na | na | 62 (12%) | 67 (14%) | 100 (18.0%) | 103 (17.5%) | 450 (19%) | 467 (20%) |
| Right coronary artery | 260 (34%) | 283 (37%) | na | na | na | na | 46 (44%) | 42 (39%) | 188 (45%) | 169 (40%) | na | na | na | na | na | na | 215 (43%) | 195 (39%) | 223 (40.1%) | 266 (45.3%) | 932 (40%) | 955 (40%) |

Data are expressed as median (interquartile range), mean ± standard deviation, or number (%).

## Supplementary Table 7: Classification of lesion complexity according to the According to the American College of Cardiology/American Heart Association criteria.

|  | BIOVASC | | Politi et al. | | SMILE | | Park et al. | | MULTISTARS AMI | | Nichita-Brendea et al | | Tarasov et al. | | Maamoun et al. | | OPTION-STEMI | | iMODERN | | Total | |
| --- | --- | --- | --- | --- | --- | --- | --- | --- | --- | --- | --- | --- | --- | --- | --- | --- | --- | --- | --- | --- | --- | --- |
|  | Immediate | Staged | Immediate | Staged | Immediate | Staged | Immediate | Staged | Immediate | Staged | Immediate | Staged | Immediate | Staged | Immediate | Staged | Immediate | Staged | Immediate | Staged | Immediate | Staged |
| N of lesions | 1569 | 1541 | na | na | 755 | 750 | na | na | na | na | na | na | na | na | na | na | na | na | na | na | 2324 | 2291 |
| Type A | 179 (11%) | 161 (10%) | na | na | 87 (12%) | 76 (10%) | na | na | na | na | na | na | na | na | na | na | na | na | na | na | 266 (11%) | 237 (10%) |
| Type B1 | 445 (28%) | 429 (28%) | na | na | 204 (27%) | 192 (26%) | na | na | na | na | na | na | na | na | na | na | na | na | na | na | 649 (28%) | 621 (27%) |
| Type B2 | 354 (23%) | 345 (22%) | na | na | 192 (25%) | 193 (26%) | na | na | na | na | na | na | na | na | na | na | na | na | na | na | 546 (23%) | 538 (23%) |
| Type C | 591 (38%) | 606 (39%) | na | na | 272 (36%) | 289 (39%) | na | na | na | na | na | na | na | na | na | na | na | na | na | na | 863 (37%) | 895 (39%) |

Data are expressed as median (interquartile range), mean ± standard deviation, or number (%).

## Supplementary Table 8: Medical treatment at discharge

|  | BIOVASC | | Politi et al. | | SMILE | | Park et al. | | MULTISTARS AMI | | Nichita-Brendea et al. | | Tarasov et al. | | Maamoun et al. | | OPTION-STEMI | | iMODERN | | Total | |
| --- | --- | --- | --- | --- | --- | --- | --- | --- | --- | --- | --- | --- | --- | --- | --- | --- | --- | --- | --- | --- | --- | --- |
|  | Immediate | Staged | Immediate | Staged | Immediate | Staged | Immediate | Staged | Immediate | Staged | Immediate | Staged | Immediate | Staged | Immediate | Staged | Immediate | Staged | Immediate | Staged | Immediate | Staged |
| N | 761 | 759 | 63 | 65 | 264 | 263 | 103 | 106 | na | na | na | na | na | na | na | na | 498 | 496 | 556 | 587 | 2245 | 2276 |
| Aspirin | 761 (100%) | 759 (100%) | 62 (98%) | 65 (100%) | 261 (99%) | 259 (98%) | 103 (100%) | 106 (100%) | na | na | na | na | na | na | na | na | 479/485 (98·8%) | 483/490 (98·6%) | 543 (97.6%) | 569 (97.0%) | 1666/1676 (99%) | 1676/1683 (100%) |
| Ticagrelor | 532 (70%) | 536 (71%) | na | na | 153 (58%) | 152 (58%) | na | na | na | na | na | na | na | na | na | na | 156/485 (32·2%) | 136/490 (27·8%) | na | na | 841/1510 (56%) | 824/1512 (54%) |
| Prasugrel | 105 (14%) | 107 (14%) | na | na | 2 (1%) | 6 (2%) | na | na | na | na | na | na | na | na | na | na | 127/485 (26·2%) | 129/490 (26·3%) | na | na | 234/1510 (15%) | 242/1512 (16%) |
| Clopidogrel | 124 (16%) | 116 (15%) | 61 (97%) | 65 (100%) | 109 (41%) | 105 (40%) | 93 (90%) | 95 (90%) | na | na | na | na | na | na | na | na | 198/485 (40·8%) | 225/490 (45·9%) | na | na | 585/1676 (35%) | 606/1683 (36%) |
| Statins | na | na | 57 (90%) | 60 (92%) | 260 (98%) | 263 (100%) | 91 (88%) | 96 (91%) | na | na | na | na | na | na | na | na | 475/485 (97·9%) | 484/490 (98·8%) | 510 (91.8%) | 544 (92.7%) | 883/915 (97%) | 903/924 (98%) |
| Beta-Blocker | na | na | 52 (83%) | 52 (80%) | 247 (94%) | 246 (94%) | 81 (79%) | 88 ( 83%) | na | na | na | na | na | na | na | na | 421/485 (86·8%) | 436/490 (89·0%) | 441 (79.3%) | 478 (81.5%) | 801/915 (88%) | 822/924 (89%) |
| RAAS-inhibitor | na | na | 35 (56%) | 38 (58%) | 256 (97%) | 249 (95%) | 74 (72%) | 78 (74%) | na | na | na | na | na | na | na | na | 449/485 (92·6%) | 453/490 (92·4%) | na | na | 814/915 (89%) | 818/924 (89%) |

Data are expressed as median (interquartile range), mean ± standard deviation, or number (%).

## Supplementary Table 9: Procedural characteristics

|  | BIOVASC | | Politi et al. | | SMILE | | Park et al. | | Multistars Ami | | Nichita-Brendea et al. | | Tarasov et al. | | Maamoun et al. | | OPTION-STEMI | | iMODERN | | Total | |
| --- | --- | --- | --- | --- | --- | --- | --- | --- | --- | --- | --- | --- | --- | --- | --- | --- | --- | --- | --- | --- | --- | --- |
|  | Immediate | Staged | Immediate | Staged | Immediate | Staged | Immediate | Staged | Immediate | Staged | Immediate | Staged | Immediate | Staged | Immediate | Staged | Immediate | Staged | Immediate | Staged | Immediate | Staged |
| N= | 764 | 761 | 65 | 65 | 264 | 263 | 103 | 106 | 418 | 422 | 50 | 50 | 67 | 69 | 36 | 42 | 498 | 496 | 556 | 587 | 2821 | 2861 |
| Three vessel disease | na | na | 19 (29%) | 29 (45%) | na | na | 17 (17%) | 22 (21%) | na | na | 13 (26 %) | 10 (20 %) | 32 (47.8%) | 31 (44.9 %) | 8 (22.2%) | 11 (26.2%) | 106 (21%) | 99 (20%) | na | na | 231/819 (28%) | 202/828 (24%) |
| Radial access | 738 (97%) | 731 (96%) | na | na | 220 (83%) | 224 (85%) | 27 (26%) | 25 (24%) | 301 (72%) | 311 (74%) | na | na | na | na | 0% | 0% | 371 (74%) | 379 (76%) | 513/553 (92.8%) | 552/585 (94.4%) | 2170/2636 (82%) | 2222/2675 (83%) |
| FFR or iFR | 118 (15%) | 177 (23%) | na | na | 65 (25%) | 71 (27%) | na | na | 12 (3%) | 36 (9%) | 5 (10 %) | 6 (12%) | na | na | na | na | na | na | 541 (97.3%) | 65 (11.1%) | 741/2052 (36%) | 355/2083 (17%) |
| Complete/succsessful revascularisation | 740 (97%) | 726 (96%) | na | na | na | na | 101 (98%) | 105 (99%) | na | na | na | na | na | na | na | na | na | na | na | na | 841/867 (97%) | 831/867 (96%) |
| OCT or intravascular ultrasonography | 45 (6%) | 111 (15%) | na | na | 70 (27%) | 69 (26%) | na | na | 10 (2%) | 15 (4%) | na | na | na | na | na | na | 118 (24%) | 139 (28%) | 52 (9.4%) | 30 (5.1%) | 295/2500 (12%) | 364/2529 (14%) |
| Index procedure duration (min) | 64 (47-84) | 45 (34-61) | na | na | 61 (38-79) | 44 (28-59) | na | na | 73 (58-93) | 52 (40-69) | na | na | na | na | na | na | 16·4 (11·3–23·1) | 9·5 (6·5–14·6) | na | na | na | na |
| Contrast use in index procedure (mL) | 200 (150-260) | 140 (100-183) | na | na | 295 (195-400) | 180 (140-230) | na | na | 250 (199-320) | 170 (130-220) | na | na | 325.8 ± 110.2 | 373 ± 154.5 | na | na | 180 (140–225) | 130 (100–180) | na | na | na | na |
| N of stents used per patient (total) | 3 (2-4) | 3 (2-4) | na | na | 3 (2-4) | 3 (2-4) | na | na | 3 (2-4) | 3 (2-4) | na | na | 2.6±0.5 | 2.7±0.6s | 2.31 ± 0.47 | 2.38 ± 0.49 | 2 (1–2) | 2 (1–2) | 2.1±1.4 | 1.8±1.1 | na | na |
| Drug-eluting stents (%) | 100% | 100% | 5/65 (8%) | 6/65 (9%) | 661/798 (83%) | 653/794 (82%) | 278/266 (100%) | 278/278 (100%) | na | na | na | na | 100% | 100% | 26/82 (31.7%) | 35/98 (35.7%) | 100% | 100% | 765/765 (100%) | 829/829 (100%) | na | na |

Data are expressed as median (interquartile range), mean ± standard deviation, or number (%).

## Supplementary Table 10: Risk of Bias Assessment according to the Revised Cochrane risk-of-bias tool for randomized trials (RoB 2)

| **Study** | Domain 1: Risk of bias arising from the randomization process | Domain 2: Risk of bias due to deviations from the intended interventions (*effect of assignment to intervention*) | Domain 2: Risk of bias due to deviations from the intended interventions (*effect of adhering to intervention*) | Domain 3: Risk of bias due to missing outcome data | Domain 4: Risk of bias in measurement of the outcome | Domain 5: Risk of bias in selection of the reported result | **Overall risk of bias** |
| --- | --- | --- | --- | --- | --- | --- | --- |
| **BIOVASC** | Low | Low | Low | Low | Some | Low | **Some risk** |
| **Politi et al.** | Some | High | Low | Low | Some | Low | **High risk** |
| **SMILE** | Low | Low | Low | Low | Some | Low | **Some risk** |
| **Park et al.** | Low | Low | Low | Low | Some | Low | **Some risk** |
| **MULTISTARS AMI** | Low | Low | Low | Low | Some | Low | **Some risk** |
| **Nichita-Brendea et al.** | Low | Low | Low | Low | Some | Low | **Some risk** |
| **Tarasov et al.** | Low | Low | Low | Low | Some | Low | **Some risk** |
| **Maamoun et al.** | Some | Some | Some | Some | Some | Some | **Some risk** |
| **OPTION-STEMI** | Low | Some | Low | Low | Some | Low | **Some risk** |
| **iMODERN** | Some | Low | Low | Low | Low | Low | **Some risk** |

## Supplementary Table 11: Assessment of level of certainty of evidence according to GRADE recommendations

| **Outcome** | | **All-cause mortality at 1 years** |
| --- | --- | --- |
| Trials | | 10 RCTs  all open label |
| Number of patients (intention-to-treat population) | | 5,651  (2,809 versus 2,842) |
| Pooled effect (95% CI) | | Risk Ratio 1.10  95% CI 0.79 to 1.52 |
| Down-grading  factors | Risk of bias | moderate |
|  | Imprecision | moderate |
|  | Inconsistency | moderate |
|  | Indirectness | moderate |
|  | Publication bias | low |
| Up-grading factors | Large magnitude of effect | no |
|  | Dose-response gradient | n.a. |
|  | All residual confounding would decrease magnitude of effect | n.a. |
| **Level of certainty** | | **MODERATE** |

# Supplementary Figures

## Supplementary Figure 1: PRISMA Flowchart of identified records

## Supplementary Figure 2: Univariate linear meta-regression analysis between the log- transformed risk ratio of all-cause mortality at 1 year and total sample size in the intention-to-treat population of each study.

Each study is represented by one circle, with the size of the circle indicating the weight of the study in the analysis. The linear regression line (continuous black line) is presented with 95% confidence intervals (blue area and dashed line). The horizontal grey line indicates a risk ratio of 1 (i.e., no effect). The studies are label as follows: 1 = iMODERN; 2 = OPTION-STEMI; 3 = MULTISTARS AMI; 4 = BIOVASC; 5 = Park et al; 6 = Nichita-Brendea et al.; 7 = Tarasov et al.; 8 = SMILE; 9 = Maamoun et al.; 10 = Politi et al.

## Supplementary Figure 3: Univariate linear meta-regression analysis between the log- transformed risk ratio of all-cause mortality at 1 year and the number of study sites of each study.

## Supplementary Figure 4: Univariate linear meta-regression analysis between the log- transformed risk ratio of all-cause mortality at 1 year and the percentage of patients with chronic hypertension.

## Supplementary Figure 5: Univariate linear meta-regression analysis between the log- transformed risk ratio of all-cause mortality at 1 year and the proportion of male participants (%) of each study.

## Supplementary Figure 6: Univariate linear meta-regression analysis between the log- transformed risk ratio of all-cause mortality at 1 year and the proportion of patients with diabetes (%) of each study.

## Supplementary Figure 7: Univariate linear meta-regression analysis between the log- transformed risk ratio of all-cause mortality at 1 year and the event rate of all-cause mortality within each study.
